# Spiral Perfusion Imaging With Consecutive Echoes (SPICE^™^) for the Simultaneous Mapping of DSC- and DCE-MRI Parameters in Brain Tumor Patients: Theory and Initial Feasibility

**DOI:** 10.18383/j.tom.2016.00217

**Published:** 2016-12

**Authors:** Eric S. Paulson, Douglas E. Prah, Kathleen M. Schmainda

**Affiliations:** 1 Departments of Radiation Oncology and; 2 Radiology, Medical College of Wisconsin, Milwaukee, Wisconsin

**Keywords:** DSC-MRI, DCE-MRI, perfusion, brain tumors, SPICE

## Abstract

Dynamic contrast-enhanced (DCE) and dynamic susceptibility contrast (DSC) magnetic resonance imaging (MRI) are the perfusion imaging techniques most frequently used to probe the angiogenic character of brain neoplasms. With these methods, *T*_1_- and *T*_2_/*T*_2_*-weighted imaging sequences are used to image the distribution of gadolinium (Gd)-based contrast agents. However, it is well known that Gd exhibits combined *T*_1_, *T*_2_, and *T*_2_* shortening effects in tissue, and therefore, the results of both DCE- and DSC-MRI can be confounded by these opposing effects. In particular, residual susceptibility effects compete with *T*_1_ shortening, which can confound DCE-MRI parameters, whereas dipolar *T*_1_ and *T*_2_ leakage and residual susceptibility effects can confound DSC-MRI parameters. We introduce here a novel perfusion imaging acquisition and postprocessing method termed Spiral Perfusion Imaging with Consecutive Echoes (SPICE) that can be used to simultaneously acquire DCE- and DSC-MRI data, which requires only a single dose of the Gd contrast agent, does not require the collection of a precontrast *T*_1_ map for DCE-MRI processing, and eliminates the confounding contrast agent effects due to contrast extravasation. A detailed mathematical description of SPICE is provided here along with a demonstration of its utility in patients with high-grade glioma.

## Introduction

Dynamic susceptibility contrast (DSC) and dynamic contrast-enhanced (DCE) magnetic resonance imaging (MRI) are the two most common contrast agent techniques used to probe the angiogenic character of brain neoplasms (1). With DSC-MRI, the *T*_2_* effects of gadolinium (Gd)-chelated contrast agents are exploited. Using this approach, a concentrated bolus of Gd, confined to the intravascular space and perfusing through a tissue capillary bed, induces transient signal loss through spin dephasing caused by vascular–extravascular susceptibility gradients (2, 3). Analysis of DSC-MRI data using indicator dilution theory provides hemodynamic estimates such as relative cerebral blood volume (rCBV), cerebral blood flow (CBF), and mean transit time (4, 5). With DCE-MRI, the *T*_1_ effect of Gd contrast agents is exploited. In particular, contrast agent extravasation, arising from disruptions of the blood–brain barrier (BBB), gives rise to signal enhancement through dipolar interaction between Gd's unpaired electrons and local tissue protons (6, 7). Pharmacokinetic analysis of DCE-MRI data provides insight into the underlying tissue pathophysiology through, for example, estimation of the blood–brain volume transfer constant (K^trans^); fractional volume of the extravascular, extracellular space (EES) (v_e_); and the efflux rate constant from EES to plasma (k_ep_) (8, 9).

Although DSC- and DCE-MRI approaches depend on the predominance of *T*_2_* and *T*_1_ effects, respectively, the results of both DSC- and DCE-MRI may be confounded by the opposing relaxation effects of Gd. For example, the shift in compartmental distribution of the contrast agent from the intravascular space to the EES can result in *T*_1_ shortening effects that, although necessary for the DCE-MRI technique, compete with and confound DSC-MRI susceptibility-induced signal decreases (10–12). The most well-characterized DSC-MRI parameter affected by *T*_1_ leakage effects is rCBV, and several DSC-MRI acquisition and analysis methods have been developed and applied to mitigate the underestimation of rCBV due to the *T*_1_ leakage effects (13–16).

In this regard, it has been shown that dual-echo acquisition methods (13, 17–21) may be one of the most robust approaches for collecting DSC-MRI data in patients with brain tumor, as *T*_1_ leakage effects can be directly eliminated (17, 22–27). However, it has also been hypothesized that residual *T*_2_/*T*_2_* effects, attributable to recirculation and/or contrast agent leakage, may result in overestimations of DSC-MRI parameters if not taken into account.

The first goal of this paper is to introduce a novel method for DSC-MRI perfusion imaging, whereby leakage effects manifesting as either *T*_1_ or *T*_2_/*T*_2_* effects can be corrected. The second goal of this paper is to demonstrate that, by using the same dual-echo spiral acquisition method, DCE-MRI parameters can be derived concurrently, independent of the precontrast calibration scans (eg, *T*_1_ maps). Consequently, the complete array of DSC- and DCE-MRI parameters, corrected for confounding contrast agent effects, can be obtained simultaneously in a single acquisition with a single dose of the Gd contrast agent. The feasibility of the method is demonstrated in patients with high-grade brain tumors.

### Theory

To motivate use and ensure full understanding of the advantages of the Spiral Perfusion Imaging with Consecutive Echoes (SPICE) approach, the theory underlying conventional DSC- and DCE-MRI in comparison with SPICE is described here.

### Derivations of DSC-MRI Concentration–Time Curves

#### Conventional DSC-MRI.

**Figure 1.**
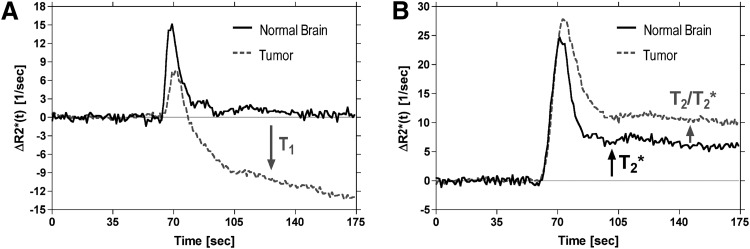
Illustration of confounding leakage and recirculation effects on dynamic susceptibility contrast (DSC)-magnetic resonance imaging (MRI) concentration–time curves for representative voxels in normal brain and brain tumor. Representative ΔR_2_^*^(t) concentration–time curves are shown for voxels in normal brain and brain tumor after serial primary (1^0^), 0.1 mmol/kg) (A) and secondary (2^0^, 0.2 mmol/kg) (B) injections of Gd contrast agent in the same patient with glioma. Acquisitions were performed at 1.5 T using a gradient echo-echo planar imaging (GRE-EPI) pulse sequence with flip angle = 90°, TE = 30 milliseconds, and repetition time (TR) = 1000 milliseconds. In regions of normal brain with an intact BBB, a concentrated bolus of Gd contrast agent will remain compartmentalized to the vasculature, resulting in transient signal changes, that ultimately return to the prebolus baseline value (A). However, in regions of tumor with a disrupted blood–brain barrier (BBB), a fraction of the contrast agent will leak out of the vasculature into the extravascular extracellular space (EES), resulting in *T*_1_ shortening effects that contaminate tumor concentration–time curves. After secondary injection, the postbolus portions of both normal brain and the tumor concentration–time curves are elevated above their prebolus baseline values (B). The fact that this occurs in normal brain, with a presumably intact BBB, suggests that this is not a leakage effect, but instead may be attributable to a residual susceptibility effect caused by recirculation of an increased steady-state concentration of the contrast agent. However, the additional elevated endline in the tumor concentration–time curve suggests a dipolar *T*_2_ leakage effect or additional susceptibility effect. These curves show that both dipolar *T*_1_ and *T*_2_ and/or residual susceptibility effects may confound perfusion estimates derived by DSC-MRI.

The concentration–time curves in DSC-MRI are generated based on an assumed linear relationship between the Gd contrast agent concentration and the change in *apparent transverse* relaxation rate induced by the first passage of the contrast agent through the vasculature (3), and it is calculated using the following equation:

(1) $$\Delta R_{2}\left(t\right)=\frac{1}{T_{2}^{*}(t)}-\frac{1}{T_{2_{0}}^{*}}\;=\kappa [Gd](t)$$

where κ is a constant dependent on transverse relaxivity, field strength, pulse sequence, and vascular morphology (2). In conventional DSC-MRI, a rapid acquisition method is used to acquire susceptibility-weighted images, and the pulse sequences typically used are of the spoiled gradient echo (GRE) family. The generalized signal equation for conventional DSC-MRI is as follows:

(2) $$S\left(t\right)=S_{0}sin\theta \left[\frac{1-e^{\frac{-TR}{T_{1}(t)}}}{1-cos\theta e^{\frac{-TR}{T_{1}(t)}}}\right]e^{\frac{-TE}{T_{2}^{*}(t)}}$$

where *T*_1_(t) and *T*_2_*(t) indicate that these parameters can change dynamically during acquisition. As described in detail in the online Supplemental Appendix [equations A1 to A5], equation 2 can be used to obtain general expressions for the pre- and postcontrast *T*_2_* values, from which a general expression for ΔR2*(t) can be derived as follows:

(3) $$\Delta R_{2}^{*}\left(t\right)=\frac{-1}{TE}\ln\left[\frac{S(t)}{\left[\frac{1-e^{\frac{-TR}{T_{1}(t)}}\;}{1-cos\theta e^{\frac{-TR}{T_{1}(t)}}}\right]}\frac{\left[\frac{1-e^{\frac{-TR}{T_{10}}}\;}{1-cos\theta e^{\frac{-TR}{T_{10}}}}\right]}{S_{B}}\right]$$

where *T*_10_ is the precontrast *T*_1_ relaxation time and S_B_ is the mean of the precontrast baseline signal determined by averaging S(t) over the first N_B_ baseline points. Equation 3 shows the potential influence of dipolar *T*_1_ effects on concentration–time curves obtained with DSC-MRI. In particular, in the absence of an intact BBB, extravasation of the contrast agent results in *T*_1_ shortening, causing a confounding reduction in ΔR_2_^*^(t) (Figure 1A).

In the presence of an intact BBB, the contrast agent remains confined to the vasculature (ie, no extravasation occurs), *T*_1_(t) is essentially equal to *T*_10_ (ie, its precontrast value), and ΔR_2_^*^(t) reduces to its ubiquitous form as follows:

(4) $$\text{Δ}R_{2}^{*}\left(t\right)=\frac{-1}{TE}\ln\left(\frac{S(t)}{S_{B}}\right)$$

### Correction of DSC-MRI Time Courses for *T*_1_ Extravasation Effects

Dual-echo acquisition methods provide an effective means by which confounding dipolar *T*_1_ leakage effects can be eliminated from DSC-MRI time courses (17–21). The signal equation for the first and second echoes (TE_i=1,2_) is as follows:

(5) $$S_{TE_{i}}\left(t\right)=S_{0}sin\theta \left[\frac{1-e^{\frac{-TR}{T_{1}(t)}}}{1-cos\theta e^{\frac{-TR}{T_{1}(t)}}}\right]e^{\frac{-TE_{i}}{T_{2}^{*}(t)}}$$

Taking the ratio of the 2 signal equations, an expression for both the baseline and postcontrast 1/*T*_2_*(t) can be derived, which is as detailed in the online Supplemental Appendix [equations A7 to A11]. From these, the change in the transverse relaxation rate can be derived as follows:

(6) $$\text{Δ}R_{2}^{*}\left(t\right)=\frac{1}{\left(TE_{2}-TE_{1}\right)}\text{ln}\left(\frac{S_{TE_{1}}\left(t\right)}{S_{TE_{2}}(t)}\;\frac{S_{TE_{2B}}}{S_{TE_{1B}}}\right)$$

Equation 6 is the DSC-MRI concentration–time curve that is free from the dipolar *T*_1_ leakage effects.

### Correction of DSC-MRI Time Courses for *T*_2_/*T*_2_* Effects

In practice, we have observed another potential confounding effect on DSC-MRI concentration–time curves characterized as elevated endlines that develop following the first pass of the contrast agent. As shown in Figure 1B, the effect appears to be exacerbated in brain tumors relative to the normal brain, which implies that there could be an additional susceptibility or *T*_2_ leakage effect in these regions beyond the effects of recirculation. The source of the elevated endlines could be dipolar *T*_2_ effects, residual susceptibility effects from the contrast agent, or some combination of both. Regardless of the source of these elevated endlines, perfusion parameters (eg, rCBV) generated using DSC-MRI may be *overestimated* if postprocessing algorithms do not account for their confounding effects (24).

**Figure 2.**
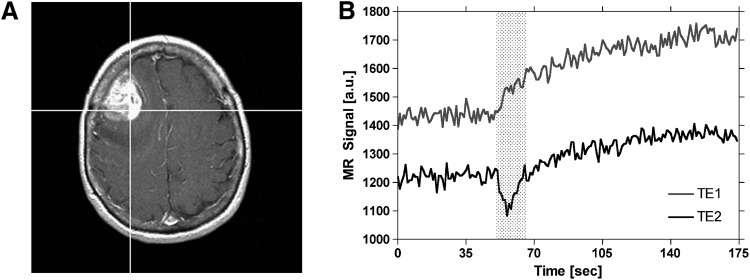
Demonstration of the time course of extravasation effects in DSC-MRI. Postcontrast *T*_1_-weighted image of a patient with a high-grade glioma (A). Spiral Perfusion Imaging with Consecutive Echoes (SPICE) signals, obtained at short and long TE, for the representative tumor voxel depicted on the postcontrast *T*_1_-weighted image (B). Leakage of contrast agent begins at the appearance time of the bolus and occurs during the first pass of the bolus (indicated by the shaded region). Following the first pass, leakage continues at a slower rate until back-diffusion occurs (not shown).

**Figure 3.**
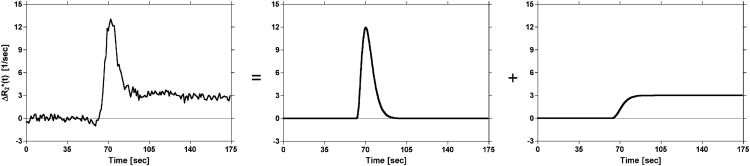
Illustration of the proposed model of DSC-MRI concentration–time curves. The representative ΔR_2_^*^(t) concentration–time curve is modeled by a γ-variate plus its cumulative integral scaled by a constant. The γ-variate is used to model the first pass of the tracer, whereas the cumulative integral is used to model recirculation and/or leakage of the tracer.

One approach discussed in the literature for analyzing DSC-MRI data is voxel-wise γ-variate fitting to the concentration–time curves (10). Although fitting of a γ-variate effectively eliminates the majority of recirculation and leakage effects that occur after the first pass, it does not remove the confounding effects of leakage that occur *during* the first pass (Figure 2). A more appropriate model of a DSC-MRI time courses with elevated endlines, introduced by Johnson et al. (28), consists of a γ-variate plus its cumulative integral as follows:

(7) $$\text{Δ}R_{2}^{*}\left(t\right)\prime =k{\left(t-t_{0}\right)}^{\alpha }e^{\frac{-\left(t-t_{0}\right)}{\beta }}+h\overset{t}{\underset{0}{\int }}k{\left(t\prime -t_{0}\right)}^{\alpha }e^{\frac{-\left(t-t_{0}\right)}{\beta }}dt\prime$$

where *k* is a scale factor, t_0_ is the appearance time of the bolus, α and β are fit parameters, and h is used to scale the cumulative integral of the γ-variate (Figure 3). Correction for elevated endlines is then performed by nonlinear least squares fitting of equation 7 to the corrupted ΔR_2_^*^(t) concentration–time curves on a voxel-wise basis. After nonlinear least squares fitting, ΔR_2_^*^(t) curves corrected for dipolar *T*_1_ and *T*_2_ and residual susceptibility effects are generated by constructing γ-variates using the parameters estimated from the full model fit as follows:

(8) $$\text{Δ}R_{2}^{*}\left(t\right)\prime =k{\left(t-t_{0}\right)}^{\alpha }e^{\frac{-\left(t-t_{0}\right)}{\beta }}$$

**Figure 4.**
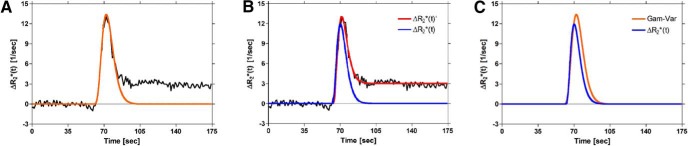
Illustration of the differences between methods used to correct DSC-MRI concentration–time curves for recirculation and/or leakage effects. γ-variate fit to ΔR_2_^*^(t) (orange) (A). Proposed full model fit to ΔR_2_^*^(t) (red) and corrected first pass (blue) (B). Corrected first-pass curves obtained from (A) and (B), (C). Compared to the standard γ-variate (orange), the corrected first-pass curve (blue) from the proposed method is characterized by decreased peak height and bolus width, which should be more representative of the actual first pass in the absence of recirculation and leakage effects.

In comparison with standard γ-variate fits, this two-step method, described by equations 7 and 8, results in corrected concentration–time curves that exhibit reduced peak height and bolus width, as expected in the absence of recirculation and leakage (Figure 4). Conventional algorithms can then be applied to generate estimates of DSC-MRI parameters that are free from confounding contrast agent effects.

### Derivation of DCE-MRI Concentration–Time Curves

#### Conventional DCE-MRI.

The concentration–time curves for DCE-MRI are generated on the basis of an assumed linear relationship between Gd concentration and the change in *spin lattice* relaxation rate, ΔR1, resulting primarily from the extravasation of the contrast agent from the vasculature to the EES, where a dipolar interaction between the unpaired electrons of the contrast agent and local tissue protons ensues (7):

(9) $$\text{Δ}R_{1}\left(t\right)=\frac{1}{T_{1}(t)}-\frac{1}{T_{1_{0}}}=R_{1}\left[Gd\right](t)$$

where $R_{1}$ is the *T*_1_ relaxivity of the Gd contrast agent. The DCE-MRI technique relies on the sensitivity of the pulse sequence to changes in signal intensity caused by *T*_1_ shortening. Traditionally, conventional 2- or 3-dimensional spoiled GRE sequences are used in DCE-MRI because they provide good image quality with sufficient temporal resolution. Analogous to DSC-MRI, the generalized signal equation for DCE-MRI is then equivalent to equation 2.

Several methods have been used to convert the dynamic signal intensity time courses into tissue Gd concentration–time curves. In the method used here [which is similar to the Hittmair approach (29)], 1/*T*_1_(t) and 1/*T*_10_ are obtained directly by solving the pre- and postcontrast signal equations as described in the online Supplemental Appendix [equations A15 and A16], and the results, along with equation 9, are used to determine ΔR_1_(t) as follows:

(10) $$\text{Δ}R_{1}\left(t\right)=\frac{-1}{TR}\text{ln}[\left[\frac{S_{0}sin\theta e^{\frac{-TE}{T_{2}^{*}(t)}}-S(t)}{S_{0}sin\theta e^{\frac{-TE}{T_{2}^{*}(t)}}-S\left(t\right)cos\theta }\right]\times \left[\frac{S_{0}sin\theta e^{\frac{-TE}{T_{2_{0}}^{*}}}-S_{B}cos\theta }{S_{0}sin\theta e^{\frac{-TE}{T_{2_{0}}^{*}}}-S_{B}}\right]]\;\;$$

Equation 10 shows the potential influence of *T*_2_* effects on the concentration–time curves obtained with DCE-MRI. In particular, *T*_2_* shortening may cause a confounding reduction in ΔR_1_(t). However, because minimum echo times (TE) are typically used, it is widely assumed that an insignificant phase dispersion will occur over time scales of short TE (ie, TE ≪ *T*_2_*). Consequently, *T*_2_* effects are generally ignored, which results in the following approximation:

(11) $$\text{Δ}R_{1}\left(t\right)\approx \frac{-1}{TR}\text{ln}\left[\left[\frac{S_{0}sin\theta -S(t)}{S_{0}sin\text{θ}-\text{S}\left(\text{t}\right)cos\theta }\right]\left[\frac{S_{0}sin\theta -S_{B}cos\theta }{S_{0}sin\theta -S_{B}}\right]\right]$$

In addition, note that because *T*_10_ is determined directly from the precontrast baseline signal intensity, equation 11 does not exhibit dependence on the initial precontrast spin lattice relaxation time. Therefore, the approach eliminates the necessity of acquiring a separate precontrast *T*_1_ map. The ΔR_1_(t) can be estimated directly from S(t), provided that an estimate of S_0_ be obtained. This is made possible by using the dual-echo SPICE sequence, as described in detail in the online Supplemental Appendix [equations A19 to A22].

### Correction of DCE-MRI Time Courses for *T*_2_/*T*_2_* Effects

Dual-echo acquisitions offer two significant advantages for DCE-MRI. One advantage is that, as discussed in the previous section, S_0_ can be determined from the first time point (ie, the first repetition) of a single-shot, dual-echo acquisition using the methodology described in the online Supplemental Appendix. This factor can result in significant time savings, in that no additional precontrast calibration scans are required to convert the DCE-MRI signal time courses into concentration–time curves. It may also improve the overall quality and accuracy of the computed parameter maps, since interscan patient motion is no longer an issue. Of potentially greater significance, it eliminates the confounding influence of spatial variations in B1 that result when images are acquired at multiple flip angles to determine precontrast *T*_1_ maps (30–32).

Another advantage of SPICE is that the confounding *T*_2_* effects of the contrast agent can be eliminated from the DCE-MRI concentration–time curves. First, 1/*T*_2_*(t) is estimated at each time point from the first and second echo signal. Second, a corrected first echo signal, S_TE1C_(t), is obtained by extrapolating each time point of the first echo signal back to TE = 0 using the following equation:

(12) $$S_{TE_{1C}}\left(t\right)=S_{TE_{1}}\left(t\right)e^{\frac{+TE_{1}}{T_{2}^{*}(t)}}=S_{0}sin\theta \left[\frac{1-e^{\frac{-TR}{T_{1}(t)}}}{1-cos\theta e^{\frac{-TR}{T_{1}(t)}}}\right]$$

Notice that *T*_2_* effects have been eliminated in the corrected signal equation. Using the TE-corrected signal at baseline **(**S_BC_) and postcontrast (S_TE1C_(t)), the ΔR_1C_(t), corrected for confounding *T*_2_* effects, can be computed using the following equation:

(13) $$\text{Δ}R_{1}\left(t\right)=\frac{-1}{TR}\text{ln}[\left[\frac{S_{0}sin\theta -S_{TE_{1C}}(t)}{S_{0}sin\theta -S_{TE_{1C}}\left(t\right)cos\theta }\right]\times \;[\frac{S_{0}sin\theta -S_{B_{C}}cos\theta }{S_{0}sin\theta -S_{B_{C}}}]]\;$$

An estimate of S_0_, determined from the first time point of the SPICE acquisition, is then substituted into equation 13, which is then used to determine the concentration–time curves using equation 9.

## Materials and Methods

The feasibility of the SPICE method was shown in two patients with tissue-confirmed high-grade glioma exhibiting enhancement on postcontrast *T*_1_-weighted images. Informed written consent was obtained from these patients under guidelines established by our Institution's Institutional Review Board.

### Data Acquisition

Images were acquired on a 1.5 T GE CV scanner (GE Healthcare, Milwaukee, Wisconsin), equipped with 40 mT/m gradients (150 T/m/s slew rate), using a commercial quadrature radiofrequency coil. Precontrast fluid-attenuated inversion recovery (FLAIR), diffusion-weighted imaging (DWI), *T*_1_, and *T*_2_ images were collected as part of the standard clinical protocol. SPICE images were then acquired using a custom, multislice 2-dimensional, single-shot, dual GRE, spiral-out sequence with the following parameters: field of view: 22 cm^2^, matrix: 96 × 96, TE_1_: 3.1 milliseconds, TE_2_: 41 milliseconds, TR: 1350 milliseconds, flip angle: 72°, slice thickness: 5 mm, skip: 1.5 mm, number of slices: 13, and number of samples (reps): 180. A 30-second delay was inserted between prescan and the beginning of the SPICE acquisition to allow full recovery of longitudinal magnetization. This facilitated estimation of the equilibrium magnetization from the first time point of the SPICE acquisition and eliminated the necessity of collecting a separate precontrast calibration scan, as described by equations A19 to A22 in the online Supplemental Appendix. A single dose of gadodiamide (0.1 mmol/kg, Omniscan®, GE Healthcare, Inc., Princeton, New Jersey) was injected at 3 mL/s using a power injector 60 seconds after the start of acquisition (33). Postcontrast *T*_1_-weighted images were then acquired as part of the standard clinical protocol (TE/TR/NEX/matrix = 11/650/2/256).

**Figure 5.**
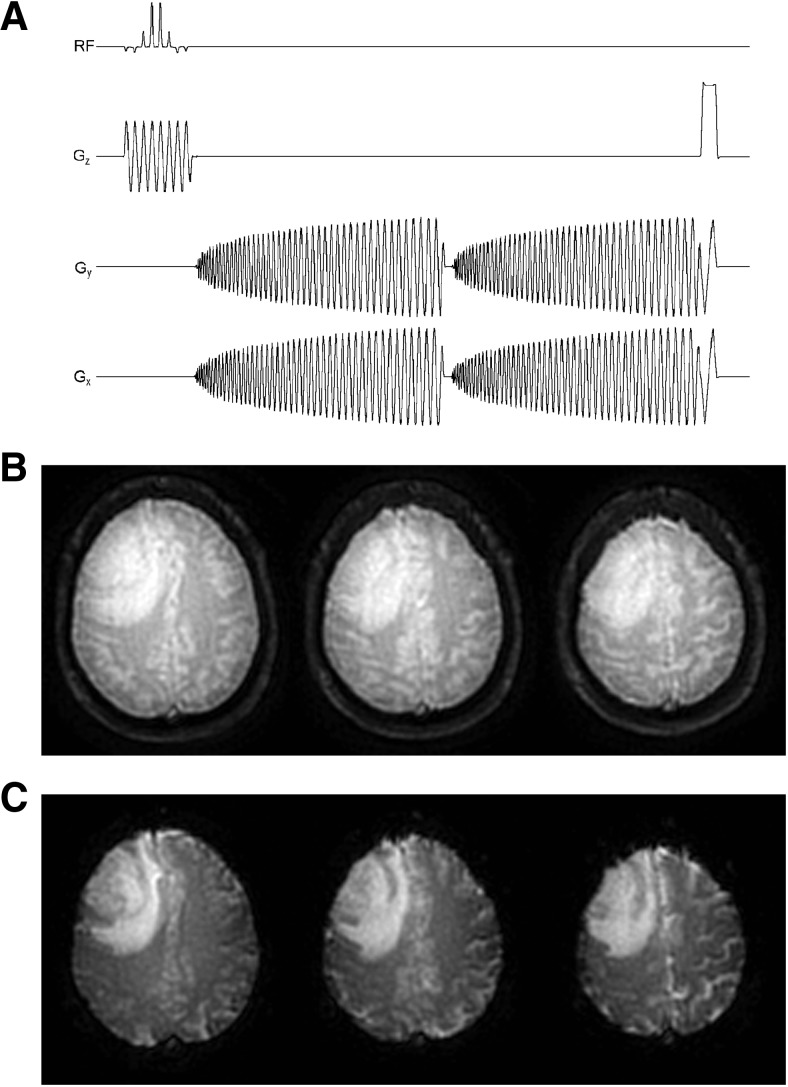
Multislice 2-dimensional single-shot, dual gradient echo (GRE), spiral-out pulse sequence (ie, SPICE) used in the present study (A). Reconstructed first (B) and second (C) echo spiral images of a patient with brain tumor acquired at 1.5 T. Images are from the first time point (ie, infinite TR) of the dual-echo acquisition. See text for acquisition parameters.

As shown in Figure 5A, SPICE acquires two echoes sequentially within a free induction decay, immediately following a spatial–spectral (SPSP) excitation pulse. The SPSP excitation pulse was used to reduce the chemical shift contributions to off-resonance effects through selective excitation of water (34). The Ernst angle (72°) was chosen to maximize the signal-to-noise ratio (SNR) of the SPICE images to prevent signal saturation at the rectified noise floor during the first passage of the contrast agent. Signal saturation can result in nonlinearities in the relationship between signal changes and contrast agent concentration, introducing an error into the estimate of the arterial input function (AIF) (35). The spiral gradient waveforms were implemented using the Glover approximation (36). For a 96 × 96 matrix, the spiral waveforms consisted of 10 863 points corresponding to a readout duration of approximately 36 milliseconds. The spiral-out direction was chosen to increase the SNR and minimize the TE of the first echo, which maximized the *T*_1_ weighting for good DCE sensitivity.

### Data Analysis

The raw SPICE data was transferred to a remote Linux workstation (quad, dual-core 2.0 GHz Opteron CPUs, 16 GB RAM, SUSE 10.2, Advanced Micro Devices, Inc., Sunnyvale, California) and reconstructed offline using custom MATLAB (Version 7.5, R2007b, The MathWorks, Inc., Natick, Massachusetts) and ANSI C software developed at our Institution. Sample-reconstructed first and second echo spiral images from the first time point of the SPICE acquisition are shown in Figure 5B–C. The reconstructed images were then postprocessed using AFNI (30) and custom software developed at our Institution.

### DSC-MRI

For comparison of SPICE with conventional methods, three versions of ΔR_2_^*^(t) concentration–time curves were generated and used in the DSC-MRI analysis:

1. ΔR_2_^*^(t) generated using only the second echo (ie, *T*_2_*-weighted) signal of the dual-echo acquisition [equation 4], similar to the conventional single-echo DSC-MRI.
2. ΔR_2_^*^(t) generated using the ratio of the SPICE dual-echo signals [equation 6, similar to previous dual-echo DSC-MRI approaches.
3. ΔR_2_^*^(t) generated using the ratio of the SPICE dual-echo signals and corrected for recirculation and any additional *T*_2_/*T*_2_* leakage effects [equation 8].

Hemodynamic parameters were estimated from the aforementioned three concentration–time curves using conventional DSC-MRI algorithms. In particular, estimates of rCBV were obtained using the following equation:

(14) $$rCBV=\frac{k_{h}\overset{\infty }{\underset{0}{\int }}\text{Δ}R_{2}^{*}\left(\tau \right)d\tau }{\rho \overset{\infty }{\underset{0}{\int }}AIF\left(\tau \right)d\tau }$$

where ρ is the density of the brain tissue (1.04 g/mL); k_h_ is a correction factor for the difference in large versus small vessel hematocrit (HCT) (4), and it is calculated as follows:

(15) $$k_{h}=\frac{1-0.45}{1-0.25}$$

AIF is the arterial input function, generated by averaging ΔR_2_^*^(t) time courses from 3 voxels manually selected in regions of the middle cerebral arteries. Estimates of CBF were then obtained from the maximum of the residue function, determined by deconvolving the tissue ΔR_2_^*^(t) curves and AIF using singular value decomposition (37). The CBF estimates were then cross-calibrated to units of absolute CBF, by scaling the mean normal-appearing white matter CBF value to 22 mL/100 mL/min (38).

### DCE-MRI

For comparison of the proposed with the conventional methods, two versions of ΔR_1_(t) concentration–time curves were generated and used in the DCE-MRI analysis:

1. ΔR_1_(t) generated using only the first echo (ie, *T*_1_-weighted) signal of the dual-echo acquisition [equation 11], similar to the conventional single-echo DCE-MRI.
2. ΔR_1_(t) generated by extrapolating the first echo signal back to TE = 0 using the dual-echo signals [equation 13]. The ΔR_1_(t) curves were then converted into tissue concentration–time curves, C_T_(t), using equation 9, giving the following equation:
  (16) $$C_{T}\left(t\right)=\left[Gd\right]\left(t\right)=\frac{\Delta R_{1}(t)}{R_{1}}$$

where $R_{1}$ is the longitudinal relaxivity of gadodiamide at 1.5 T (∼4.39 s^−1^mM^−1^ at 37°C) (39). A surrogate for the plasma concentration–time curve, C_p_(t), was determined in a 2-step process. First, the tissue concentration–time curves for three (M = 3) manually selected voxels containing arteries were averaged to determine an arterial concentration–time curve, Ca(t) as follows:

(17) $$C_{a}\left(t\right)=\frac{1}{M}\overset{M}{\underset{j=1}{\sum }}{\left(C_{T}(t)\right)}_{j}$$

Second, the arterial concentration–time curve was adjusted for HCT to produce the plasma concentration–time curve as follows:

(18) $$C_{p}\left(t\right)=\frac{C_{a}(t)}{\left(1-HCT\right)}$$

where an assumed value of 0.45 was used for HCT (40). Pharmacokinetic analysis of DCE-MRI data was then performed using conventional algorithms. In particular, the volume transfer constant between blood plasma and EES, K^trans^, and the fractional volume of the plasma space, v_p_, was determined on a voxel-by-voxel basis by linear least squares fitting of the linearized Patlak model to the tissue and plasma concentration–time curves (41) as follows:

(19) $$C_{T}\left(t\right)=K^{trans}\overset{t}{\underset{0}{\int }}C_{p}\left(t\prime \right)dt\prime =v_{p}C_{p}(t)$$

## Results

**Figure 6.**
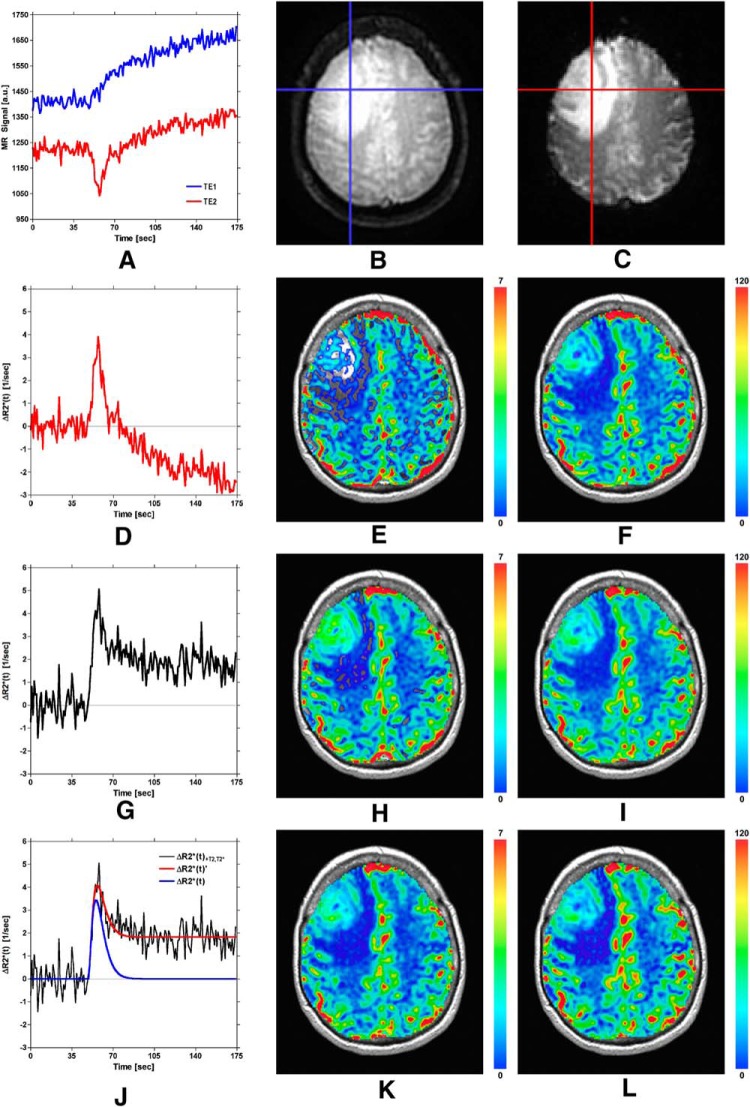
Demonstration of correction of DSC-MRI data using the proposed SPICE postprocessing method. See text for details.

The effect of correcting DSC-MRI concentration–time curves for confounding recirculation and leakage is shown in Figure 6. Figure 6A displays the dual-echo time series for the representative tumor voxel depicted on the first and second echo spiral images shown in Figure 6B–C. Note that the signals have been truncated to remove the first few points during which the signal approached a steady state. Extravasation of the contrast agent is apparent from the increase in signal intensity shown on both the first and second echo signals. Because the first echo signal (ie, the blue curve) is heavily *T*_1_-weighted, the leakage effect is apparent as an immediate signal increase. However, because the second echo signal (ie, the red curve) is more strongly *T*_2_*-weighted, a transient signal decrease is observed, with the signal increase becoming apparent after the initial transient. By comparing the dual-echo signals, note that the leakage of the contrast agent begins at the appearance time of the bolus, occurs during the first pass of the bolus, and continues after the first pass of the bolus.

Figure 6D–F displays the ΔR_2_^*^(t) curve (for the same tumor voxel) obtained from the second echo (ie, *T*_2_*-weighted) signal only [equation 4], similar to conventional single-echo DSC-MRI, along with corresponding rCBV and CBF maps. Note that the curve in Figure 6D is confounded by *T*_1_ leakage effects, which causes the postbolus ΔR_2_^*^(t) to fall below the prebolus baseline and results in an underestimation of rCBV. This effect is apparent by a lack of blood volume (ie, regions of transparency) in Figure 6E, which is exacerbated in tumor regions.

Figure 6G–I displays the ΔR_2_^*^(t) curve (for the same tumor voxel) obtained from the ratio of the dual-echo signals [equation 6], similar to previous dual-echo approaches, along with corresponding rCBV and CBF maps. By using the ratio of the dual-echo signals when constructing ΔR_2_^*^(t), confounding *T*_1_ effects are eliminated, resulting in an increased peak height of ΔR_2_^*^(t) relative to that shown in Figure 6D and the unmasking of the recirculation and *T*_2_/*T*_2_* leakage effects (evident from the elevated endline/postbolus baseline). The elimination of *T*_1_ effects prevents underestimation of rCBV and CBF, evident by comparing Figure 6, H and I with Figure 6, E and F.

Figure 6J–L displays representative ΔR_2_^*^(t)' (red) and ΔR_2_^*^(t) (blue) curves obtained using equations 7 and 8, along with corresponding rCBV and CBF maps. Note that, after the proposed correction, the blue curve shown in Figure 6J and rCBV and CBF maps in Figure 6, K and L are no longer confounded by recirculation or by any dipolar *T*_1_ and *T*_2_ and/or residual susceptibility leakage effects. The corrected rCBV map in Figure 6K shows reduced rCBV values relative to Figure 6H (most notably in the tumor). This suggests that an overestimation of rCBV can result in the presence of recirculation and any residual susceptibility or dipolar *T*_2_ leakage effects. Although the proposed correction also reduced CBF values in Figure 6L relative to Figure 6I, the reduction is minimal compared with rCBV.

**Figure 7.**
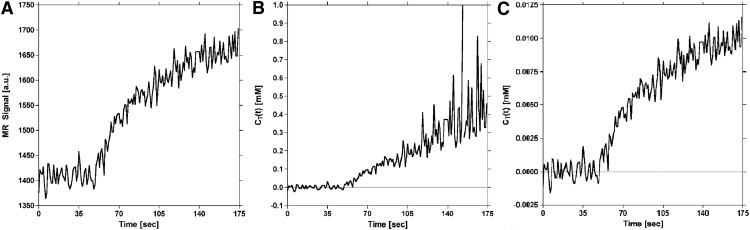
Influence of S_0_ estimates on dynamic contrast-enhanced (DCE)-MRI tissue concentration–time curves generated with the proposed method. *T*_1_-weighted (first-echo) signal time course for a representative voxel in tumor (A). Calculated tissue concentration–time curve for tumor voxel in (A) generated using S_0_ estimated *without* full recovery of longitudinal magnetization (B). With increasing signal enhancement (ie, as the signal approaches S_0_), the noise in the concentration–time curve is amplified (note the scale on the ordinate). Calculated tissue concentration–time curve for tumor voxel in (A) generated using S_0_ estimated *with* full recovery of longitudinal magnetization (C). Allowing full recovery of longitudinal magnetization results in a concentration–time curve shape that closely resembles the signal in (A), prevents amplification of noise during signal enhancement, and reduces error.

Figure 7 shows the influence of S_0_ estimates on DCE-MRI concentration–time curves constructed using equation 13. As shown in Figure 7B, failure to wait long enough for full recovery of longitudinal magnetization between prescan and the start of the acquisition results in an underestimation of S_0_ and amplified noise when the increase in signal intensity due to extravasation approaches the underestimated value of S_0_. However, by allowing full recovery of longitudinal magnetization, noise amplification is prevented, resulting in a concentration–time curve profile (Figure 7C) that matches the signal time course (Figure 7A).

**Figure 8.**
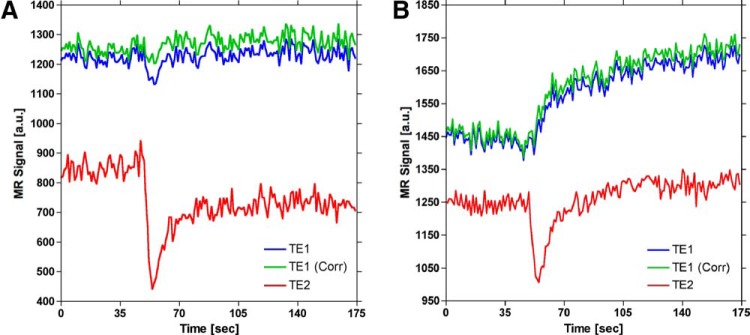
Demonstration of confounding *T*_2_* effects on signals used to generate DCE-MRI concentration–time curves for representative voxels in (A) artery and (B) tumor. The dual-echo signals (blue and red curves) are used to extrapolate the first-echo signal back to TE = 0 (green curve), which eliminates the influence of *T*_2_* effects. During the first pass of a bolus injection of a contrast agent, *T*_2_* effects can confound *T*_1_-weighted signals (blue curve), which can introduce error in DCE-MRI arterial input functions (AIFs). Although the majority of confounding *T*_2_* effects are probably masked by *T*_1_ shortening, correction for *T*_2_* effects in tumor results in an apparent increased rate of signal enhancement, which can influence heuristic DCE-MRI signal analysis (B).

Figure 8 shows the influence of *T*_2_* effects on DCE-MRI time courses. Figure 8A displays the first, corrected first, and second echo signals for a voxel in an artery. A transient signal decrease is observed in both first echo (ie, *T*_1_-weighted) and second echo (ie, *T*_2_*-weighted) time series. As shown by the corrected signal (ie, green curve) in Figure 8A, the magnitude of the *T*_2_* signal decrease is reduced using the dual-echo signals to extrapolate the first echo signal back to TE = 0 millisecond. In addition, residual susceptibility effects due to recirculation, evident from the postbolus portion of the second echo signal remaining below its prebolus baseline, are also recovered in the corrected signal. Figure 8B displays the first, corrected first, and second echo signals for a voxel in tumor. Correction for *T*_2_* effects resulted in a slight increase in the rate of signal enhancement over the entire postbolus region.

**Figure 9.**
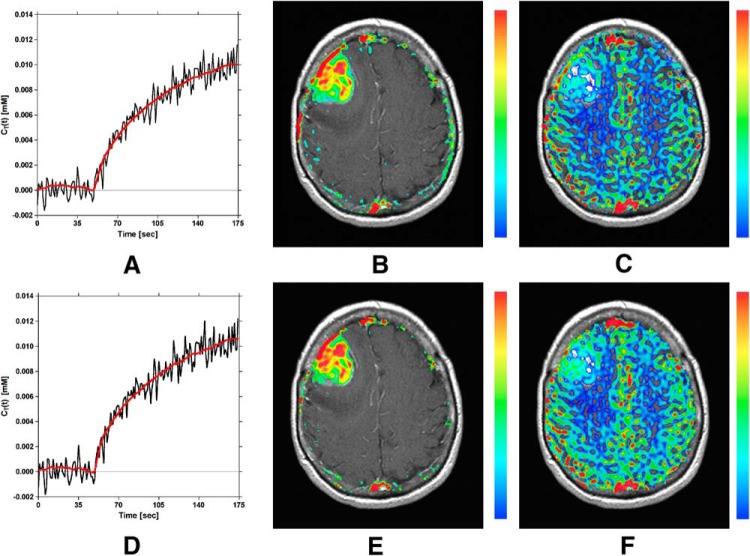
Demonstration of the correction of DCE-MRI data using the proposed SPICE method. Top row: Linear least squares Patlak model fit (red line) to tissue concentration–time curve (A), and corresponding estimates of K^trans^ (B) and v_p_ (C). The concentration–time curve in (A) was constructed using a single echo signal time course analogous to conventional DCE analysis. Second row: Linear least squares Patlak model fit (red line) to tissue concentration–time curve (D), and corresponding estimates of K^trans^ (E) and v_p_ (F). The concentration–time curve in (D) was constructed using a dual-echo corrected signal time course, which facilitated correction for *T*_2_*(t) effects.

The effects of correcting DCE-MRI concentration–time curves for *T*_2_* effects are shown in Figure 9. Figure 9A–C displays a representative tissue concentration–time curve (A), along with corresponding K^trans^ (B) and v_p_ (C) maps, generated using only the first echo signal time course analogous to conventional DCE-MRI analysis [equation 13]. Figure 9D–F displays the tissue concentration–time curve (D), along with corresponding K^trans^ (E) and v_p_ (F) maps, for the same voxel as Figure 9A, but corrected for confounding *T*_2_* effects using the dual-echo signal time courses to extrapolate the first echo signal back to TE = 0 millisecond [ie, equation 15]. In both cases, the DCE-MRI parameters were obtained following linear least squares fitting of the Patlak model fit (red line) to the tissue concentration–time curves (black lines in Figure 9, A and D). Only slight spatial differences in the K^trans^ and v_p_ maps are apparent by comparing Figure 9, E and F with Figure 9, B and C.

## Discussion

We have presented the mathematical theory and feasibility of SPICE, a spiral-based perfusion imaging method by which DSC- and DCE-MRI perfusion imaging data can be derived simultaneously, with high temporal resolution using only a single dose of contrast agent. This approach has several distinct advantages over the more common approach of obtaining DSC and DCE data separately and with different imaging sequences. In particular, by using a spiral-based approach, which encodes two echoes simultaneously within an free induction decay (FID), both *T*_1_-weighted (short TE) and *T*_2_*-weighted (longer TE) data can be obtained with a temporal resolution of about 1 second. Although it was previously shown that a temporal resolution of close to 1 second is best to obtain the most accurate DCE parameter estimations (17, 42), such resolution cannot be achieved with the standard fast GRE methods commonly used to collect DCE-MRI data. Therefore, the dual-echo GRE spiral sequence may represent a significant step forward in achieving more robust and reproducible DCE parameters. In turn, this could translate into greater standardization across patients and sites, which has been a long-standing goal of DCE perfusion imaging.

Another important advantage of the SPICE approach is that a preload of the contrast agent is no longer necessary to diminish the contrast agent leakage effects as previously recommended when using single-echo DSC methods (11, 12, 15, 43, 44). Therefore, all data can be obtained using only a single dose of the contrast agent. This advantage is of particular importance given the recent restrictions implemented by the Food and Drug Administration on the use of Gd-based agents because of the small but real risk of nephrogenic systemic fibrosis (45) and more recent concerns regarding Gd deposition in brain (46, 47).

A further advantage of using the SPICE approach is that separate precontrast S_0_ and *T*_1_ calibration scans, traditionally required for DCE-MRI analysis (33, 48, 49), are not required. Eliminating the need for these additional scans reduces the total scan time and several potential errors associated with the collection of additional precontrast calibration scans. For example, when using multiple flip angle methods to determine the precontrast *T*_1_, incomplete spoiling of transverse coherence can cause large errors in the determination of *T*_1_ that vary with the choice of TR and flip angle (50). In addition, the potential for errors due to interscan patient motion and B1 field inhomogeneities can be precluded by eliminating this step. Finally, with SPICE, the DSC-MRI parameters are implicitly corrected for *T*_1_ leakage effects, and both DSC- and DCE-MRI parameters can be corrected for residual susceptibility effects and *T*_2_/*T*_2_* effects arising from contrast agent recirculation and leakage. Consequently, this approach has the potential to provide the most accurate and comprehensive array of MRI perfusion parameters.

Despite the many demonstrated and potential advantages of this approach, there remain several aspects that need further study and optimization. For example, the 1350-millisecond TR used in this paper was chosen to obtain greater brain coverage while also maintaining a temporal resolution close to 1 second. A drawback of the longer interimage TR (relative to standard DCE TRs) is a reduction in the *T*_1_ weighting, which may not be optimal for DCE parameter estimates. Although a longer TR decreases *T*_1_ weighting, this problem diminishes at higher field strengths, which are being increasingly used. Future work will include implementation of parallel transmit capabilities to improve slice coverage while minimizing TR for improved *T*_1_ contrast (51).

Although the SPICE method does not require estimation of a precontrast *T*_1_ map, one must take into account dependence of this estimate on the SNR and a number of precontrast baseline points sampled in the DCE acquisition. Poor SNR and a small number of precontrast baseline points could affect the accuracy of the baseline signal estimate, and thus the initial *T*_1_ estimate. In the current implementation, a flip angle of 72° (the Ernst angle) and 60 baseline points were acquired to maximize SNR and thus improve the accuracy of the precontrast baseline signal intensity. Future studies to characterize these dependencies are planned.

An additional practical requirement, to ensure the collection of high-quality baseline signal intensities, is that sufficient time elapses between the performance of the prescan and the start of scanning. As shown in Figure 7, poor-quality baseline signal will result if scanning immediately follows the prescan. In this work, the scanner operator waited 30 seconds between the end of prescan and beginning acquisition, an overly conservative estimate of the time needed to allow full recovery of longitudinal magnetization. A more robust approach may be to use 30 seconds worth of discarded acquisitions (ie, disdaqs) with 0° flip angle. This would be one approach to ensure that the time between prescan and scanning is sufficient for full relaxation of the longitudinal magnetization and any potential variations in delays between scanner operators are eliminated.

The spiral-based approach has another potential option for easily determining the precontrast *T*_1_. Theoretically, it is possible to estimate a *T*_1_ map directly from the signal transients obtained at the beginning of the perfusion-weighted imaging time series. However, the short *T*_1_ at 1.5 T and a rather coarse temporal sampling of 1350 milliseconds used in the current implementation preclude this because there are not enough points to adequately fit a curve and produce robust estimates of the initial *T*_1_. However, this approach may find utility at higher fields or with shorter TRs.

In this study, correction for residual susceptibility effects was performed using the model introduced by Johnson et al. (28). It should be emphasized that although this model is based on the γ-variate function, the approach is not equivalent to the γ-variate fitting performed in many studies to determine rCBV from ΔR2*(t). Rather, it uses the cumulative integral of the γ-variate function to fit the recirculation effects, which are subsequently corrected. Also, in the context of fitting and correcting residual DSC baseline effects, this approach does not attempt to distinguish contributions because of recirculation from those resulting from the contrast agent leakage. Given that a residual DSC signal baseline is often apparent in a normal-appearing brain, residual DSC signal baselines observed in tumor cannot be attributed entirely to contrast agent leakage effects. Thus, there is no clear alterative at this time but to fit and correct the residual baseline with a lumped-fitting approach, as is used here.

A comparison between the proposed method and the established DCE methods is necessary, although beyond the scope of this paper. Current DCE methods use conventional spoiled GRE sequences (eg, spoiled gradient recalled echo [SPGR] or fast low angle shot magnetic resonance imaging [FLASH]) for data acquisition. The effective TR for these methods is roughly 6–15 seconds even though it has been shown that the DCE signal time course should be sampled about every 1 second for the most accurate parameter estimations (49, 52). The proposed method offers a reduced TR and should improve AIF quality. However, a direct comparison between the more accepted conventional DCE methods and the new DCE method proposed here should be undertaken.

In this work, the Patlak model was used to estimate DCE parameters, K^trans^, and v_p_. A more comprehensive approach would be to use the extended Tofts model to estimate K^trans^, kep, and ve. However, only 3 minutes of SPICE data were collected such that in some voxels, the washout phase of the contrast agent was not observed, thereby precluding the use of the extended Tofts model. Future studies will extend the temporal sampling of the SPICE data from 3 to 7 minutes so that the models can be compared. Although the Patlak model was used for pharmacokinetic analysis, other DCE-MRI models could be readily applied.

The necessity for correcting DCE time courses for *T*_2_* effects may be questioned, given the short TEs used in conventional DCE methods and in the proposed method. However, as shown in Figure 8, *T*_2_* may also affect large vessels (eg, AIF) and tumor vasculature may contain vessels with a distribution of radii, resulting in more or less confounding effects from *T*_2_*. Also, the differences in slopes shown in Figure 9, though seemingly small, suggest that heuristic DCE-MRI analysis methods may benefit from *T*_2_* correction. The need for this step will be further explored with the planned DCE comparison studies described above. Nevertheless, even if *T*_2_* effects are negligible, dual-echo acquisitions still permit conversion of signal intensity time courses into concentration–time curves without the need to acquire a precontrast *T*_1_ map.

The spiral-based approach described here offers several advantages that make it well-suited for perfusion imaging. Unlike echo planar imaging (EPI), spiral imaging does not collect data in the corners of k-space, resulting in increased time efficiency over EPI. The shorter readout durations in spiral translate into several advantages, including reduced *T*_2_* decay during the readout, which limits the maximum achievable resolution of single-shot methods (53–55); increased temporal resolution, which is beneficial for AIF sampling in DSC and DCE imaging (52, 56–58), or increased *T*_1_-weighting in DCE (59); increased section coverage for a given TR; and diminished vessel blooming (17). Specific to DCE-MRI, because the readout starts in the center of the k-space, spirals can achieve very short minimum TEs, producing images with good *T*_1_ weighting.

A major disadvantage of spiral is compromised image fidelity because of off-resonance-induced phase accrual over the readout. The current implementation of the proposed method does not correct for off-resonance effects. It has been well established that off-resonance effects can degrade the fidelity of spiral images. In contrast to EPI, where off-resonance effects result in a dominant one-dimensional distortion along the phase-encode direction, a two-dimensional blurring results in spiral images (60). Although an SPSP pulse was used to diminish the chemical shift contributions to off-resonance effects, off-resonant spins still arise from field inhomogeneity and tissue susceptibility differences. Although the proposed method does acquire dual echoes at each slice location, the difference in TEs is very large. The large delta TE results in phase images with multiple phase wraps, requiring unwrapping of the phase images. Methods to reduce off-resonance effects include, selectively exciting water using SPSP excitation pulses to reduce the chemical shift contribution to off-resonance effects; reducing field inhomogeneity by careful shimming; and applying off-resonance correction algorithms (51, 61, 62). Parallel imaging (eg, spiral SENSE) (63) would also provide substantial benefits for the single-shot, dual-echo spiral acquisition described here by reducing the length of the spiral readout. This would greatly improve data quality in regions of static susceptibility differences, such as resection cavities. Finally, although blurring results in a local resolution loss, it does not force the requirement to spatially remap displaced pixel data to restore its actual anatomic location (55).

The proposed SPICE method requires a single dose of the contrast agent to obtain both DSC- and DCE-MRI parameters. It should be emphasized, however, that at least a single dose (ie, 0.1 mmol/kg) must be used. Although satisfactory contrast enhancement can be obtained using only a half dose of high *T*_1_ relaxivity agents such as MultiHance (Bracco Diagnostics Inc), a half-dose does not produce appropriate susceptibility effect for DSC, regardless of the method used for acquisition (ie, single- or dual-echo, EPI, or spiral) or field strength. Contrast-to-noise is critical for adequate nonlinear least squares fitting of the model to correct for *T*_2_/*T*_2_* effects and least squares fitting of the Patlak model for DCE analysis. Therefore, although the proposed method reduces the total amount of contrast agent that needs to be administered, a minimum of a single dose is highly recommended.

### Supplemental Materials

Supplemental Appendix:
