# Supplementary material for: Spiral Perfusion Imaging With Consecutive Echoes (SPICE™) for the Simultaneous Mapping of DSC- and DCE-MRI Parameters in Brain Tumor Patients: Theory and Initial Feasibility
Source: Tomography. 2016 Dec;2(4):295–307. doi: 10.18383/j.tom.2016.00217 (PMC5226659; doi:10.18383/j.tom.2016.00217)
Supplement: Supplemental Appendix: [file tom-00217-16-s001.pdf]

APPENDIX:

Derivations of DSC-MRI Concentration-Time Curves

Conventional DSC-MRI

The concentration-time curves in DSC-MRI are generated based on an assumed linear relationship between gadolinium concentration and the change in *apparent transverse* relaxation rate:

$$\Delta R_2(t) = \frac{1}{T_2^*(t)} - \frac{1}{T_{2_0}^*} = \kappa[Gd](t) \quad \text{(A1)}$$

where κ is a constant dependent on transverse relaxivity, field strength, pulse sequence, and vascular morphology (3).

The generalized signal equation for conventional DSC-MRI is:

$$S(t) = S_0 \, sin\theta \left[\frac{1 - e^{\frac{-TR}{T_1(t)}}}{1 - cos\theta \, e^{\frac{-TR}{T_1(t)}}}\right] e^{\frac{-TE}{T_2^*(t)}} \quad \text{(A2)}$$

where $T_1(t)$ and $T_2^*(t)$ indicate that these parameters can change dynamically during acquisition. An expression for $1/T_2^*(t)$ is obtained by inverting Eq. A2:

$$\frac{1}{T_2^*(t)} = \frac{-1}{TE} ln \left[\frac{S(t)}{S_0 \, sin\,\theta \left[\frac{1 - e^{\frac{-TR}{T_1(t)}}}{1 - cos\theta \, e^{\frac{-TR}{T_1(t)}}}\right]}\right] \quad \text{(A3)}$$

In order to determine the change in apparent transverse relaxation rate (i.e., $\Delta R_2^*(t)$) an estimate of the pre-contrast apparent transverse relaxation rate (i.e., $T_{2_0}^*$) must also be obtained. First, the pre-contrast baseline signal, S_B, is determined by averaging S(t) over the first N_B baseline points:

$$S_B = \frac{1}{N_B} \sum_{i=1}^{N_B} \left[S_0 \, sin\theta \left[\frac{1 - e^{\frac{-TR}{T_{10}}}}{1 - cos\theta \, e^{\frac{-TR}{T_{10}}}}\right] e^{\frac{-TE}{T_{2_0}^*}}\right] \quad \text{(A4)}$$

Note that because the contrast agent has not yet been administered, constant initial values of T_{10} and $T_{2_0}^*$ are used in the expression. Second, substituting S_B for S(t) and $T_{2_0}^*$ for $T_2^*(t)$ in Eq. A2, an expression for $1/T_{2_0}^*$ can be obtained. Finally, substituting the expressions for $1/T_2^*(t)$ and $1/T_{2_0}^*$ into Eq. A1 gives:

$$\Delta R_2^*(t) = \frac{-1}{TE} \ln \left[\frac{S(t)}{\left[\frac{1 - e^{\frac{-TR}{T_1(t)}}}{1 - cos\theta \, e^{\frac{-TR}{T_1(t)}}}\right]} \frac{\left[\frac{1 - e^{\frac{-TR}{T_{10}}}}{1 - cos\theta \, e^{\frac{-TR}{T_{10}}}}\right]}{S_B}\right] \quad \text{(A5)}$$

Eq. A5 demonstrates the potential influence of dipolar T_1 effects on concentration-time curves obtained with DSC-MRI. In the presence of an intact BBB, the contrast agent remains confined to the vasculature

(i.e., no extravasation occurs), $T_1(t)$ is essentially equal to T_{10} (i.e., its pre-contrast value), and $\Delta R_2^*(t)$ reduces to its ubiquitous form:

$$\Delta R_2^*(t) = \frac{-1}{TE}\ln\left(\frac{S(t)}{S_B}\right) \quad \text{(A6)}$$

Correction of DSC-MRI Time Courses for T_1 Extravasation Effects

Dual-echo acquisition methods provide an effective means by which confounding dipolar T_1 leakage effects can be eliminated from DSC-MRI time courses (17-21). The signal equations for the first (TE_1) and second (TE_2) echoes are:

$$S_{TE_1}(t) = S_0 \, sin\theta \left[\frac{1 - e^{\frac{-TR}{T_1(t)}}}{1 - cos\theta e^{\frac{-TR}{T_1(t)}}}\right] e^{\frac{-TE_1}{T_2^*(t)}} \quad \text{(A7)}$$

and

$$S_{TE_2}(t) = S_0 \, sin\theta \left[\frac{1 - e^{\frac{-TR}{T_1(t)}}}{1 - cos\theta e^{\frac{-TR}{T_1(t)}}}\right] e^{\frac{-TE_2}{T_2^*(t)}} \quad \text{(A8)}$$

respectively. Taking the ratio of the two signal equations, an expression for $1/T_2^*(t)$ can be obtained:

$$\frac{1}{T_2^*(t)} = \frac{1}{(TE_2 - TE_1)} \, ln\left(\frac{S_{TE_1}(t)}{S_{TE_2}(t)}\right) \quad \text{(A9)}$$

Again, the pre-contrast apparent transverse relaxation rate (i.e., $1/T_{2_0}^*$) must be estimated in order to determine the change in apparent transverse relaxation rate. Following the methodology described in the previous section, an expression for $1/T_{2_0}^*$ is obtained from the ratio of the baseline signals:

$$\frac{1}{T_{2_0}^*} = \frac{1}{(TE_2 - TE_1)} \, ln\left(\frac{S_{TE_{1B}}}{S_{TE_{2B}}}\right) \quad \text{(A10)}$$

Substituting the results of Eq. A9 and A10 into Eq. A1, we obtain the relationship:

$$\Delta R_2^*(t) = \frac{1}{(TE_2 - TE_1)} \, ln\left(\frac{S_{TE_1}(t)}{S_{TE_2}(t)} \frac{S_{TE_{2B}}}{S_{TE_{1B}}}\right) (48) \quad \text{(A11)}$$

Eq. A11 is the DSC-MRI concentration-time curve free from dipolar T_1 leakage effects.

Correction of DSC-MRI Time Courses for T_2/T_2 Effects*

In practice, we have observed another potential confounding effect on DSC-MRI concentration-time curves apparent as elevated endlines, which develop following the first pass of contrast agent, an effect due to recirculation and/or contrast agent leakage effects. To correct the corrupted $\Delta R_2^*(t)$ concentration-time curves, the data are fit on voxel-wise basis to a gamma-variate plus its cumulative integral, as introduced by Johnson et al (24):

$$\Delta R_2^*(t)' = k(t - t_0)^\alpha \, e^{\frac{-(t-t_0)}{\beta}} + h \int_0^t k(t' - t_0)^\alpha \, e^{\frac{-(t-t_0)}{\beta}} dt' \quad \text{(A12)}$$

where k is a scale factor, t_0 is the appearance time of the bolus, alpha, and beta are fit parameters, and h is used to scale the cumulative integral of the gamma-variate. After non-linear least squares fitting, $\Delta R_2{}^*(t)$ curves corrected for dipolar T_1 and T_2 and residual susceptibility effects are generated by constructing gamma-variates using the parameters estimated from the full model fit:

$$\Delta R_2^*(t)' = k(t - t_0)^{\alpha}\, e^{\frac{-(t-t_0)}{\beta}} \tag{A13}$$

Conventional algorithms can then be applied to generate estimates of DSC-MRI parameters from these corrected time courses.

Derivations of DCE-MRI Concentration-Time Curves

Conventional DCE-MRI

The concentration-time curves in DCE-MRI are generated based on an assumed linear relationship between gadolinium concentration and the change in *spin-lattice* relaxation rate:

$$\Delta R_1(t) = \frac{1}{T_1(t)} - \frac{1}{T_{1_0}} = \mathfrak{R}_1[Gd](t) \tag{A14}$$

Analogous to DSC-MRI, the generalized signal equation for DCE-MRI is then equivalent to that in Eq. A2. Then, the $1/T_1(t)$ and $1/T_{10}$ are obtained directly by solving the pre- and post-contrast signal equations and the results, along with Eq. A14, are used to determine $\Delta R_1(t)$. To begin, $1/T_1(t)$ is obtained by inverting Eq. A2:

$$\frac{1}{T_1(t)} = \frac{-1}{TR}\, ln\left[\frac{S_0 sin\theta\, e^{\frac{-TE}{T_2^*(t)}} - S(t)}{S_0 sin\theta\, e^{\frac{-TE}{T_2^*(t)}} - S(t) cos\theta}\right] \tag{A15}$$

In order to determine the change in spin lattice relaxation rate (i.e., $\Delta R_1(t)$), an estimate of the pre-contrast spin lattice relaxation rate (i.e., T_{10}) must be obtained. Following the methodology described in the previous sections, an expression for $1/T_{10}$ is obtained by inverting the pre-contrast baseline signal, S_B, constructed by averaging S(t) over the first N_B baseline points:

$$\frac{1}{T_0} = \frac{-1}{TR}\, ln\left[\frac{S_0 sin\theta\, e^{\frac{-TE}{T_2^*(t)}} - S_B}{S_0 sin\theta\, e^{\frac{-TE}{T_{2B}^*}} - S_B cos\theta}\right] \tag{A16}$$

Substituting Eq. A15 and A16 into Eq. A14:

$$\Delta R_1(t) = \frac{-1}{TR} ln\left[\left[\frac{S_0 sin\theta\, e^{\frac{-TE}{T_2^*(t)}} - S(t)}{S_0 sin\theta\, e^{\frac{-TE}{T_2^*(t)}} - S(t) cos\theta}\right]\left[\frac{S_0 sin\theta\, e^{\frac{-TE}{T_{20}^*}} - S_B cos\theta}{S_0 sin\theta\, e^{\frac{-TE}{T_{20}^*}} - S_B}\right]\right] \tag{A17}$$

Eq. A17 demonstrates the potential influence of $T_2{}^*$ effects on concentration-time curves obtained with DCE-MRI. Specifically, $T_2{}^*$ shortening will cause a confounding reduction in $\Delta R_1(t)$. However, since

minimum echo times are used in DCE-MRI to obtain good T_1 weighting, it is widely assumed that insignificant phase dispersion will occur over time scales of short TE (i.e., TE << T_2*). Consequently, T_2* effects are generally ignored, which results in the following approximation:

$$\Delta R_1(t) \approx \frac{-1}{TR} ln \left[\left[\frac{S_0 sin\theta - S(t)}{S_0 sin\theta - \text{S(t)} cos\theta} \right] \left[\frac{S_0 sin\theta - S_B cos\theta}{S_0 sin\theta - S_B} \right] \right] \quad \text{(A18)}$$

Note that since T_{10} is determined directly from the pre-contrast baseline signal intensity, **Eq. A18 does not exhibit a dependence on the initial, pre-contrast spin lattice relaxation time**. **Therefore, this approach eliminates the necessity of acquiring a separate pre-contrast T_1 map**. Furthermore, notice that $\Delta R_1(t)$ can be estimated directly from S(t), provided that an estimate of S_0 be obtained.

Assuming fully relaxed spins, S_0 can be estimated from a single-shot, single repetition (i.e., infinite TR), dual gradient-echo acquisition. In the limit that TR → ∞, the signal equations for the first and second echoes (i.e., Eq. A7 and A8) reduce to:

$$S_{TE_{10}} = S_0 sin\theta e^{\frac{-TE_1}{T_{2_0}^*}} \quad \text{(A19)}$$

and

$$S_{TE_{20}} = S_0 sin\theta e^{\frac{-TE_2}{T_{2_0}^*}} \quad \text{(A20)}$$

Using the same methodology that was used to generate Eq. A10, $1/T2_0$* is estimated as:

$$\frac{-1}{T_{2_0}^*} = \frac{1}{(TE_2 - TE_1)} ln\left(\frac{S_{TE_{10}}}{S_{TE_{20}}}\right) \quad \text{(A21)}$$

Substituting Eq. A21 into Eq. A19, S_0 is estimated as:

$$S_0 = \frac{S_{TE_{10}}}{sin\theta} e^{\frac{TE_1}{(TE_2 - TE_1)} ln\left(\frac{S_{TE_{10}}}{S_{TE_{20}}}\right)} \quad \text{(A22)}$$

The estimate of S_0 is then substituted into Eq. A18 to obtain the change in spin lattice relaxation rate, which is then used to determine the concentration-time curves using Eq. A14.

Correction of DCE-MRI Time Courses for T2/T2 Effects*

Confounding T_2* effects of the contrast agent can be eliminated from the DCE-MRI concentration-time curves when collecting SPICE data. First, $1/T_2$*(t) is estimated at each time point from the first and second echo signal using Eq. A9. Second, a corrected first echo signal, S_TE1_C(t) is obtained by extrapolating each time point of the first echo signal in Eq. A8 back to TE=0 using:

$$S_{TE_{1C}}(t) = S_{TE_1}(t) \, e^{\frac{+TE_1}{T_2^*(t)}} = S_0 sin\theta \left[\frac{1 - e^{\frac{-TR}{T_1(t)}}}{1 - cos\theta e^{\frac{-TR}{T_1(t)}}} \right] \quad \text{(A23)}$$

Notice that T_2* effects have been eliminated in the corrected signal equation. Using the methodology used to generate Eq. A15, an expression for $1/T_1(t)$ can be obtained:

$$\frac{-1}{T_1(t)} = \frac{-1}{TR} ln\left(\frac{S_0 sin\theta - S_{TE_1}(t)}{S_0 sin\theta - S_{TE_{1C}}(t) cos\theta}\right) \tag{A24}$$

In order to determine the change in spin lattice relaxation rate (i.e., $\Delta R_1(t)$), an estimate of the pre-contrast spin lattice relaxation rate (i.e., $T1_0$) must be obtained. Following the methodology described in the previous sections, an expression for $1/T_{10}$ is obtained by inverting the pre-contrast baseline signal, S_{BC}, constructed by averaging S(t) over the first N_B baseline points:

$$\frac{1}{T_0} = \frac{-1}{TR}\, ln\left[\frac{S_0 sin\theta - S_{B_C}}{S_0 sin\theta - S_{B_C} cos\theta}\right] \tag{A25}$$

Substituting Eq. A24 and A25 into Eq.A14:

$$\Delta R_1(t) = \frac{-1}{TR} ln\left[\left[\frac{S_0 sin\theta - S_{TE_{1C}}(t)}{S_0 sin\theta - S_{TE_{1C}}(t) cos\theta}\right]\left[\frac{S_0 sin\theta - S_{B_C} cos\theta}{S_0 sin\theta - S_{B_C}}\right]\right] \tag{A26}$$

Eq. A26 is the $\Delta R_1(t)$ curve corrected for confounding T_2* effects. An estimate of S_0, determined from the first time point of the single-shot, dual-echo acquisition using Eq. A22, is then substituted into Eq. A26 to obtain the change in spin lattice relaxation rate, which is then used to determine the concentration-time curves from which the perfusion parameters are determined.
